# T Cell Production of IFNγ in Response to TLR7/IL-12 Stimulates Optimal B Cell Responses to Viruses

**DOI:** 10.1371/journal.pone.0166322

**Published:** 2016-11-23

**Authors:** Kira Rubtsova, Anatoly V. Rubtsov, Kalani Halemano, Sam X. Li, John W. Kappler, Mario L. Santiago, Philippa Marrack

**Affiliations:** 1 Howard Hughes Medical Institute, Denver, Colorado, 80206, United States of America; 2 Department of Biomedical Sciences, National Jewish Health and Department of Immunology and Microbiology, University of Colorado Health Sciences Center, Denver, Colorado 80206, United States of America; 3 Department of Medicine, University of Colorado Denver, Aurora, Colorado 80045, United States of America; 4 Department of Immunology and Microbiology, University of Colorado Denver, Aurora, Colorado 80045, United States of America; 5 Department of Pharmacology, University of Colorado School of Medicine, Aurora, Colorado 80045, United States of America; 6 University of Colorado School of Medicine, Aurora, Colorado 80045, United States of America; 7 Department of Biochemistry and Molecular Genetics, University of Colorado Health Sciences Center, Aurora, Colorado 80045, United States of America; Centre d'immunologie de Marseille Luminy, FRANCE

## Abstract

Knowledge of the processes that underlie IgG subclass switching could inform strategies designed to counteract infections and autoimmunity. Here we show that TLR7 ligands induce subsets of memory CD4 and CD8 T cells to secrete interferon γ (IFNγ) in the absence of antigen receptor stimulation. In turn, TLR ligation and IFNγ cause B cells to express the transcription factor, T-bet, and to switch immunoglobulin production to IgG2a/c. Absence of TLR7 in T cells leads to the impaired T-bet expression in B cells and subsequent inefficient IgG2a isotype switching both in vitro and during the infection with Friend virus in vivo. Our results reveal a surprising mechanism of antiviral IgG subclass switching through T-cell intrinsic TLR7/IL-12 signaling.

## Introduction

Toll-like receptors (TLRs) are pattern recognition receptors (PRRs), that are responsible for detection of microbial and viral pathogens and for induction of innate immune responses. Moreover, TLRs also influence adaptive immune responses, [1, 2] and this property has been linked to expression of TLRs on B and T cells [3, 4]. In particular, TLR expression by B cells has been shown to affect B cell responses [1, 5, 6]. The role of TLR expression in T cells has been more controversial [3, 4], but recent studies provided evidence that T cell-intrinsic TLR signaling modulates T cell responses [3, 4, 7]. These include the findings that, in LCMV-infected mice, T-cell intrinsic MyD88 (Myeloid Differentiation factor 88) expression is required for the expansion of virus-specific CD8 T cells [8, 9] and that, during *Toxoplasma gondii* infection, TLR signaling in T cells was demonstrated to be necessary for prolonged resistance to the pathogen [10]. Similarly, MyD88 signaling in CD4 T cells promotes IFNγ production in response to the intracellular bacteria *Ehrlichia muris* [11] and ablation of MyD88 in mouse T cells impaires Th17 and Th1 responses in an IL-1-dependent manner [12]. The last of these studies concluded that IL-1 induced MyD88 signaling rendered CD4 T cells refractory to Treg cell-mediated suppression. Overall, these studies demonstrate that TLRs are expressed on different T cell subsets and can modulate the response of these subsets in various ways.

One critical function of CD4 T cells is to provide help to B cells thus promoting effective humoral immune responses. However, despite the accumulated data on TLR signaling in T cells, the effect of this phenomenon on humoral immunity has not been studied. The experiments described herein were designed to address this gap in our knowledge. In previous studies, we demonstrated that synergistic stimulation of B cells through TLRs on the B cells themselves plus their antigen receptor (BCR) and their IFNγ receptor led to T-bet expression and IgG2a/c (referred to as IgG2a in the rest of this manuscript) isotype switching in the targeted B cells [13]. T-bet expressing B cells were detected in gammaherpesvirus-infected mice at the peak of the anti-viral humoral immune response and these T-bet+ B cells were crucial for effective viral clearance [13]. Thus, T-bet induction in B cells was critical for anti-viral immunity. In addition, T-bet+ B cells were detected in autoimmune mice and humans indicating that they may play a role in the induction of autoimmunity [14–16].

In our previous study involving various TLR agonists, TLR7 stimulation induced the highest amounts of IFNγ production by splenic non-B cells and hence, in the presence of anti-BCR antibodies, induced the greatest amount of T-bet expression in co-cultured B cells. However, the splenic cell type(s) that responded to TLR7 ligation by IFNγ production remained unclear. Here we report that memory CD4 and CD8 T cells respond to TLR7 triggering in IL-12 dependent manner, by IFNγ production. We show that T-cell derived IFNγ is critical for the appearance of T-bet+ B cells and IgG2a antibodies. Finally, we provide evidence that this mechanism is required for an effective anti-viral humoral immune response.

## Materials and Methods

### Mice

C57BL/6, MyD88^fl/fl^, LCK^CRE^, TLR7-/-, B6.SJL, IL-18-/- and CD19^CRE^ mice were purchased from The Jackson Laboratory and bred at the National Jewish Health animal facility. T-bet^fl/fl^ mice were generously provided by Dr. L. Glimcher. Female 6–16 weeks old mice were used for all experiments, all mice were sacrificed using CO_2_. All animals were handled in strict accordance with good animal practice as defined by the relevant national and/or local animal welfare bodies, and all animal work was approved by the National Jewish Health Animal Care and Use Committee (IACUC). The protocol was approved by National Jewish IACUC (protocol number AS2517).

### Generation of bone marrow chimeras

Bone marrow cells were isolated from C57BL/6 (WT), TLR7-/-, or TCRβ-/- mice. The C57BL/6, or TLR7-/- bone marrow cells were mixed with bone marrow from TCRβ-/- mice at 1:4 proportions and 5 x 106 cells were intravenously injected into lethally irradiated (900 rad) C57Bl/6 mice. Mice were rested for 8 weeks before any other manipulations were performed.

### In vitro cultures

Unseparated splenocytes were cultured at 5x10^6^ cells/ml at various conditions as indicated. TLR agonists were used as follows (chosen based on our previous experience): the TLR7 agonist R848 (Invivogen), at 1 μg/ml; the TLR2 agonist, Pam3Cys, at 250ng/ml; the TLR3 agonist, Poly I:C at 50 μg/ml; the TLR4 agonist, LPS, at 20 μg/ml and the TLR9 agonist, ODN1668 (Invivogen), at 1 μg/ml. IL-12 (p70) (Biolegend) was used at 20 ng/ml, and anti-IL-12 p40 clone C17.8 (eBioscience) at 20μg/ml. “Anti-B cell receptor BCR” was (Fab’)2 anti-IgM (Jackson Immune Research) and was used at 5 μg/ml.

### Flow cytometry and cell sort

Cells were stained with antibodies to mouse CD4 (clone GK1.5), CD8 (clone 53–6.7), B220 (clone RA3-6B2), CD11c (clone N418), CD19 (clone 1D3) CD44 (clone IM7), CD45.1 (clone A20), CD45.2 (clone 104), CD62L (clone MEL-14). For intracellular IFNγ staining, cells were cultured at 5 x 10^6^ cells/ml for 18h after what Golgi Plug was added for another 5h. Cells were washed, surface stained, fixed with permiabilization/fixation buffer (eBioscience) and stained with in-house made anti-IFNγ antibodies. Cells were analyzed by flow cytometry on a CyAn (Beckman-Coulter) instrument and data were analyzed using FlowJo software (Treestar).

For CD4 and CD8 naïve and memory T cells were sorted using flow sorter, splenocytes were surface stained as indicated and sorted on Synergy (Sony), post-sort purity was checked and was greater than 98% for each sorted population. B cells were isolated as CD43-neagtive fraction of splenocytes using anti-CD43 microbeads (Miltenyi Biotech).

### ELISA

Plates were coated with goat anti-mouse total IgG antibodies (Jackson labs). Supernatant IgG was detected with AP-conjugated goat anti-mouse IgG1, IgG2b, IgG2c, IgG3 or total IgG (Jackson Immune Research) as indicated. For IFNγ detection, BD OptEIA mouse IFN-γ ELISA Sets (BD) was used according to the manufacturer’s suggestions. To measure antibodies against Friend Virus (FV) ELISA plates were coated with FV lysate prepared as previously described [17]. Serum IgG was detected with biotin-conjugated goat anti-mouse IgG2c (SouthernBiotech) followed by HRP-conjugated streptavidin (SouthernBiotech).

### Friend virus infection

Mice were infected i.v. with 10^4^ spleen focus-forming units of B-tropic FV stock containing only F-MuLV and SFFV (also referred to as ‘LDV-free FV’ in prior publications) [18]. FV stocks were prepared and titered in BALB/c mice as described [19]. Spleens and serum were harvested at indicated time points after infection.

### Statistics

Data were analyzed with Prism 5 (GraphPad Software) using 2-tailed Student’s t tests. Graphs show the mean +/- SEM of the results. *, p<0.05, **, p<0.001, ***, p<0.0001

## Results

### A subset of memory T cells secretes IFNγ upon TLR7 stimulation

**Fig 1:**
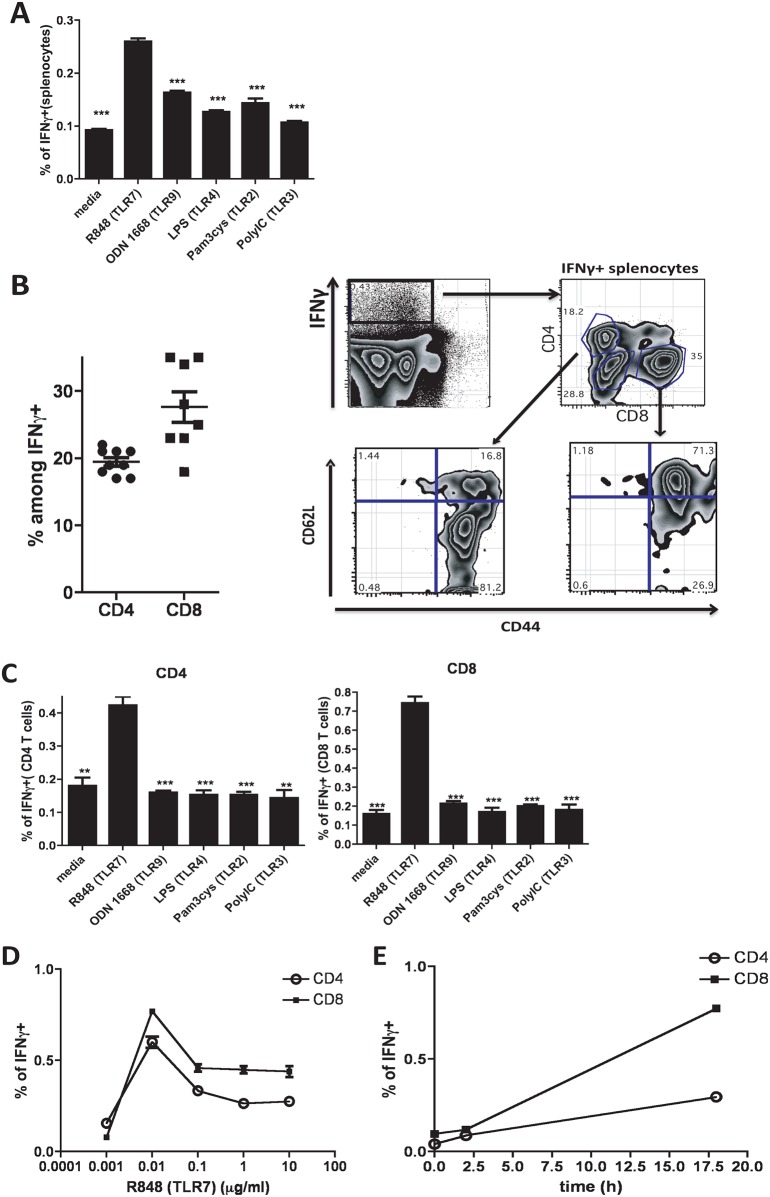
Subsets of CD4 and CD8 memory T cells produce IFNγ in response to TLR7 simulation *in vitro*. Splenocytes from C57Bl/6 mice were cultured *in vitro* for 18h in the presence of different TLR agonists (as indicated). Cells were stained for surface markers and intracellular IFNγ. **(A)** Bars represent percentage of total splenocytes positive for intracellular IFNγ. **(B)** Gating strategy for IFNγ+ splenocytes upon TLR7 simulation for 18h (Representative FACS plots) and quantification of CD4 and CD8-positive cells among IFNγ+ splenocytes. **(C)** Bar graphs represent percentages of IFNγ+ among CD4 or CD8 T cells after 18h of splenocytes stimulation with different TLR ligands as indicated. **(D, E)** Percantages of IFNγ+ CD4 and CD8 T cells in response to different doses **(D)** or different time **(E)** of stimulation with R848. Bars represent the means +/- SEM (n = 3). All data are representative of three or more independent experiments. Statistics is shown for each condition over R848 stimulated cultures.

We recently demonstrated that, of all the TLRs, engagement of TLR7 induced the highest production of IFNγ by mouse spleen cells [13]. At that time we showed that B cells were not the origin of the cytokine. Here we studied this issue in greater depth. First, we confirmed that an agonist for TLR7, compared with agonists for other TLRs, is indeed the most potent inducer of IFNγ by spleen cells. Thus, the percentage of IFNγ+ splenocytes was highest in TLR7 stimulated cultures compared to splenocytes stimulated with other TLR ligands (Fig 1A). To characterize the IFNγ producing cells, we stimulated the cells with R848 (TLR7 agonist) for 18h and then stained them as described for their surface markers and intracellular IFNγ (Fig 1B). Very few B cells and no NK cells were IFNγ+ (data not shown). However, more than 60% of the IFNγ+ cells were CD4 or CD8 positive, suggesting that T cells were the major source of the cytokine (Fig 1B). The IFNγ+ T cells did not bear Tfh or Treg markers (data not shown). However, all the IFNγ+ T cells expressed high levels of CD44, indicating a memory phenotype (Fig 1B).

There are two major subsets of memory T cells, effector and central memory, which can be distinguished by expression of CD62L (high on central memory and low on effector memory cells). The majority of the CD4+ IFNγ+ cells lacked CD62L expression, whereas CD8+ IFNγ+ cells were mostly CD62L+ (Fig 1B). These data indicate that the CD4 and CD8 cells that secrete IFNγ in response to TLR7 agonists were contained in the effector and central memory pools, respectively. Importantly, intracellular staining confirmed that CD4 and CD8 T cells produced IFNγ only in response to TLR7 engagement but not to other TLR agonists (Fig 1C). A dose response and time course analysis showed that T cells respond to low doses of R848 (starting at 0.01μg/ml) (Fig 1D) and that IFNγ production increases with time after addition of the TLR7 agonist to spleen cell cultures, with no IFNγ detected at 0h or 2h time points (Fig 1E). Together, these data demonstrate that memory CD4 and CD8 T cell subsets produce IFNγ in response to TLR7 stimulation even in the absence of T cell receptor (TCR) engagement. Moreover, the T cells produced IFNγ only in response to TLR7 but not to other TLR ligands.

### TLR7 agonists act directly on T cells to induce IFNγ production

**Fig 2:**
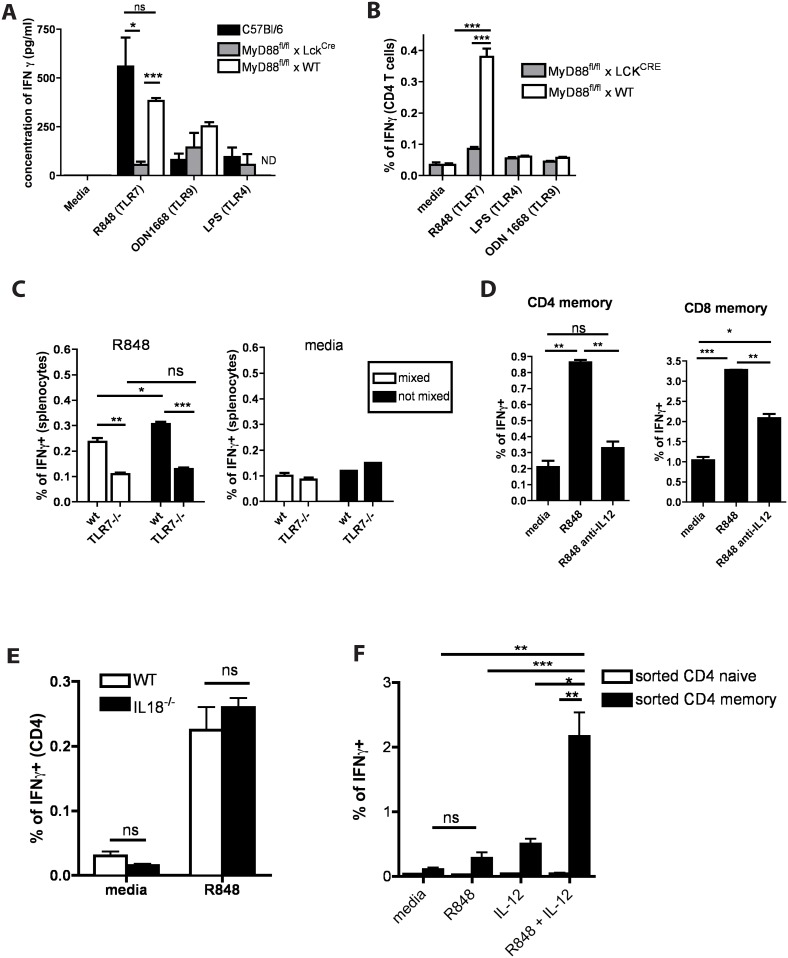
TLR7 agonist in the presence of IL-12 stimulates T cells directly leading to IFNγ production. **(A)** Spleen cells obtained from C57BL/6, MyD88^fl/fl^xLCK^CRE^ and MyD88^fl/fl^ x WTmice were incubated in the presence of different TLR agonists as indicated for 5 days. Supernatants were analyzed for the presence of IFNγ by ELISA. Bar graphs represent concentration of IFNγ in the culture supernatants. **(B)** Spleen cells from MyD88^fl/fl^ xWT or MyD88^fl/fl^xLCK^CRE^ mice were incubated in the presence of different TLR agonists as indicated for 18h. Cells were surface stained and intacellularly stained for IFNγ. Bar graphs represent percentage of CD4 or CD8 T cells which are positive for IFNγ. **(C)** TLR7^-/-^ or conjenically marked WT (B6.SJL) splenocytes were incubated either separately (black bars) or mixed at 1:1 ratio in the presence (R848) or absence (media) of TLR7 agonist. IFNγ production was assessed by intracellular staining and the summary of three independent experiments is shown. Bars represent the means +/- SEM. **(D)** Splenocytes were incubated as indicated for 18h, IFNγ production by CD4 and CD8 T cells in response to indicated stimulations was assessed by intracellular staining. Bar graphs indicate percentage of IFNγ+ cells among CD4 and CD8 T cells. **(E)** WT or IL-18^-/-^ splenocytes were incubated with R848 for 18h, IFNγ production was assessed by intracellular staining and the summary of three independent experiments is shown. Bars represent the means +/- SEM (similar results were obtained for CD8 T cells—not shown). **(F)** Naïve and memory CD4 and CD8 T cells were flow sorted as CD4 (or CD8) positive, CD19^-^, CD44+ (for memory) and CD44^-^ (for naïve). IFNγ production by sorted T cells in response to indicated stimulations was assessed by intracellular staining. Bar graph represent percentage of IFNγ+ sorted memory of naïve CD4 T cells (similar data was obtained for CD8 T cells—not shown). All data are representative of three or more independent experiments.

All the experiments described above involved cultures of unseparated spleen cells, therefore there are two possible explanations for the ability of T cells to produce IFNγ in response to TLR7 agonists: either T cells respond directly to the agonists, or some other splenic cell detects the TLR7 agonist and produces material that subsequently acts on the T cells (a bystander effect). To distinguish between these two possibilities, we used spleen cells from mice with a T cell-specific MyD88 deletion (generated by intercrossing MyD88^fl/fl^ and LCK^Cre^ mice) (S1 Fig). We stimulated splenocytes from C57Bl/6, MyD88^fl/fl^ x LCK^Cre^ mice or MyD88^fl/fl^ littermate controls with R848 (TLR7 agonist), ODN1668 (TZLR9 agonist) or LPS (TLR4 agonist) and measured the levels of IFNγ in the culture supernatants by ELISA and in T cells by intracellular cytokine staining. The cultures that contained MyD88 deficient T cells did not produce IFNγ in response to TLR7 triggering (Fig 2A) and the T cells in these cultures did not stain intracellularly for IFNγ (Fig 2B). These data indicate that memory T cells produce IFNγ following TLR7 stimulation via T cell intrinsic MyD88 signaling.

MyD88 is an adaptor molecule involved in the transduction of signals from TLRs (except TLR3) and the receptors for cytokines such as IL-1 and IL-18. A recent [12] and some older studies [20, 21] demonstrated that IL-1 could play a role in T cell activation. Thus, it is possible that the IFNγ-producing T cells in the TLR7-stimulated culture responded to IL-1 and/or IL-18 produced by other cells, rather than to the TLR7 agonist itself.

To investigate this phenomenon and, in particular, to avoid the possibility of an indirect effect on the T cells, we cultured a mixture of splenocytes obtained from TLR7-/- (CD45.1+) and B6.SJL (CD45.2+) mice at a 1:1 ratio. The mixtures were stimulated with media or R848 and IFNγ production was assessed. As demonstrated in Fig 2C, only TLR7 sufficient T cells were able to produce IFNγ upon TLR7 ligation. Thus, even in the presence of factors produced in response to TLR7 by other components in the spleen cells, the TLR7-/- memory T cells could not produce IFNγ demonstrating that they themselves had to detect the TLR7 agonist in order to respond.

The combination of IL-12 and IL-18 is known to induce IFNγ production from memory CD8 T cells [22, 23] and the IL-18 receptor signals via MyD88 which, as demonstrated above (Fig 2A and 2B) is critical for IFNγ production upon TLR7 triggering, we checked whether these cytokines play a role in this process. First, we tested the requirement for IL-12. C57Bl/6 (WT) splenocytes were stimulated with R848 in the presence or absence of anti-IL-12 blocking antibodies. As demonstrated in Fig 2D, IL-12 blockade significantly reduced the percentage of IFNγ producing T cells. Next we tested whether IL-18 was also involved. Anti-IL-18 antibodies had no effect on induction of IFNγ producing T cells by a TLR7 agonist (data not shown). In an additional test for the role of IL-18, spleen cells from WT or IL-18-/- mice were cultured without or with a TLR7 agonist. As demonstrated in Fig 2E, the TLR7 agonist induced equal amounts of IFNγ producing T cells from both WT and IL-18-/- splenocytes. Collectively these results show that IL-12 but not IL-18 is needed in order for T cells to produce IFNγ in response to a TLR7 agonist.

To confirm the role of IL-12, produced by non-T cells, we isolated naïve or memory CD4 or CD8 T cells to high purity by FACS sorting and cultured them either in medium alone, or with R848 or a combination of R848 and IL-12. As demonstrated in Fig 2F, sorted memory, but not naïve, CD4 T cells were able to produce IFNγ in response to TLR7 ligation in the presence of IL-12. The response in the absence of IL-12 was much smaller, conforming that the TLR7 agonist induced IL-12 production by non-T cells. (Similar results were obtained for sorted CD8 T cells—data not shown). This data indicate that the combination of TLR7 agonist and IL-12 is necessary and sufficient for IFNγ production by memory CD4 or CD8 T cells.

Altogether, these data indicate that IFNγ production by T cells following TLR7 simulation occurred through T-cell intrinsic TLR7/IL-12 signaling.

### IFNγ produced by memory TLR7-sufficient T cells induce T-bet expression in B cells in response to TLR7 triggering

We previously demonstrated that, following TLR7 stimulation, splenocyte-derived IFNγ and B cell receptor (BCR) crosslinking synergized to induce T-bet expression in B cells [13].

**Fig 3:**
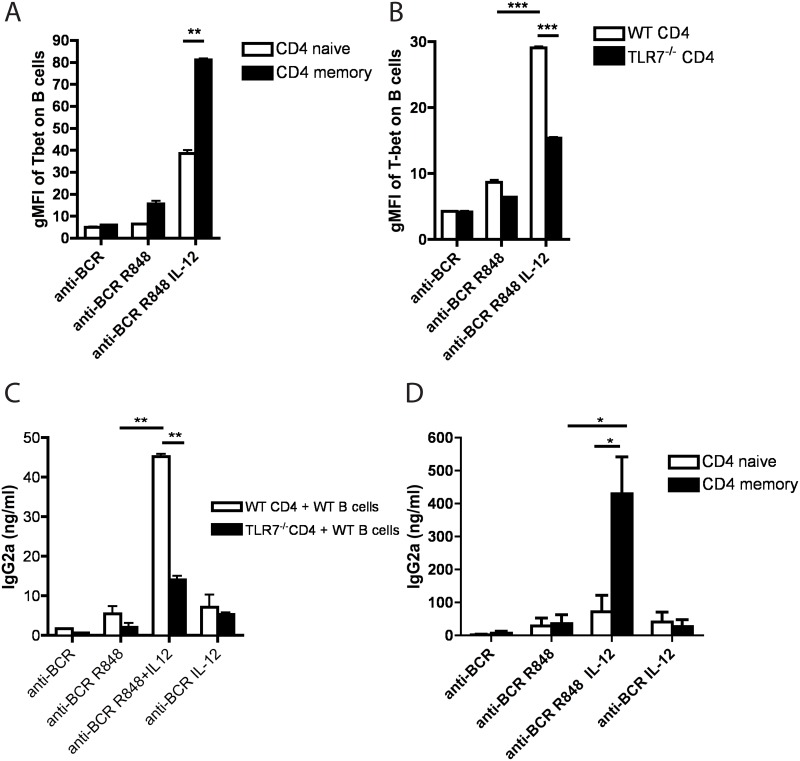
IFNγ produced by T cells in response to TLR7/IL-12 simulation is required for T-bet induction in B cells. **(A)** Sorted memory or naïve CD4 T cells were mixed with purified naïve B cells and incubated in the presence of anti-BCR, anti-BCR and R848, or combination of anti-BCR, R848 and IL-12 for 48h. Cells were stained for surface markers and intracellular T-bet. Bar graph represents gMFI of T-bet expression in B cells (gated as live, B220+, CD19+, CD4-, CD8-). Bars represent the means +/- SEM. **(B)** WT or TLR7-/- sorted CD4 T cells were mixed with purified naïve WT B cells and incubated for 48h with indicated stimuli. Cells were stained for surface markers and intracellular T-bet. Bar graph represents gMFI of T-bet expression in B cells (gated as live, B220+, CD19+, CD4-, CD8-). Bars represent the means +/- SEM **(C)** WT or TLR7-/- sorted CD4 T cells were mixed with purified naïve WT B cells and incubated in the presence of indicated stimuli for 7 days. Culture supernatants were assessed for the presence IgG2a by ELISA. **(D)** Sorted memory or naïve CD4 T cells were mixed with purified naïve B cells and incubated in the presence of indicated stimuli for 7 days. Supernatants were analyzed for the presence of IgG2a by ELISA. Bars represent the means +/- SEM. Data are representative of three or more independent experiments.

We tested the ability of highly pure sorted naïve or memory CD4 T cells to facilitate T-bet induction in B cells. As demonstrated in Fig 3A, CD4 memory were able to induce significantly higher levels of T-bet expression in B cells compared to naïve T cell, when stimulated with anti-BCR and R848 in the presence of IL-12. In order to confirm that TLR7 expression on T cells is required for T-bet induction in B cells we repeated the experiment using WT or TLR7-/- sorted T cells. Fig 3B demonstrates that WT T cells induce significantly higher levels of T-bet expression in B cells in the presence of anti-BCR, R848 and IL-12, compared to TLR7-/- T cells.

Overall, the data indicate that IFNγ production by T cells in response to TLR7/IL-12 stimulation contributes to the induction of high levels of T-bet expression in B cells.

### IFNγ produced by memory TLR7-sufficient T cells is required for IgG2a class switching upon TLR7 triggering

Our group and others established that T-bet expression in B cells is required for efficient class-switching to IgG2a [13, 24–26]. We therefore investigated whether TLR7/IL-12 responsiveness of memory T cells is required for IgG2a isotype switching. WT B cells were mixed with WT or TLR7-/- sorted T cells in the presence of anti-BCR, R848 and IL-12. As demonstrated in Fig 3C, WT but not TLR7-/-T cells were able to induce efficient IgG2a isotype switching in B cells in the presence of R848/IL-12. Furthermore this effect was largely due to the activity of memory rather than naïve T cells since, as demonstrated in Fig 3D, memory T cells induced significantly higher titers of IgG2a production compared to naïve T cells in the presence of R848 and IL-12 (IgG1 titers were similar both cultures, data not shown).

These data correlate well with the finding that TLR7 induced IFNγ production leads to T-bet induction in B cells, which in turn leads to IgG2a isotype switching.

### TLR7 expression in T cells is required for the appearance of T-bet+ B cells and anti-viral IgG2a production during Friend virus infection

So far we have demonstrated that memory CD4 and CD8 T cells produce IFNγ following TLR7 stimulation in IL-12 dependent manner. Moreover, IFNγ produced by T cells in response to TLR7/IL-12 triggering is required for efficient induction of T-bet in B cells and their subsequent switch to IgG2a production. We previously demonstrated that T-bet expression in B cells is critical for anti-viral IgG2a production and effective viral clearance [13]. Since IgG2a is known to be the most efficient IgG subclass for viral clearance [27, 28] we investigated whether TLR7 expression in T cells plays a role in B cell responses to viral infection.

**Fig 4:**
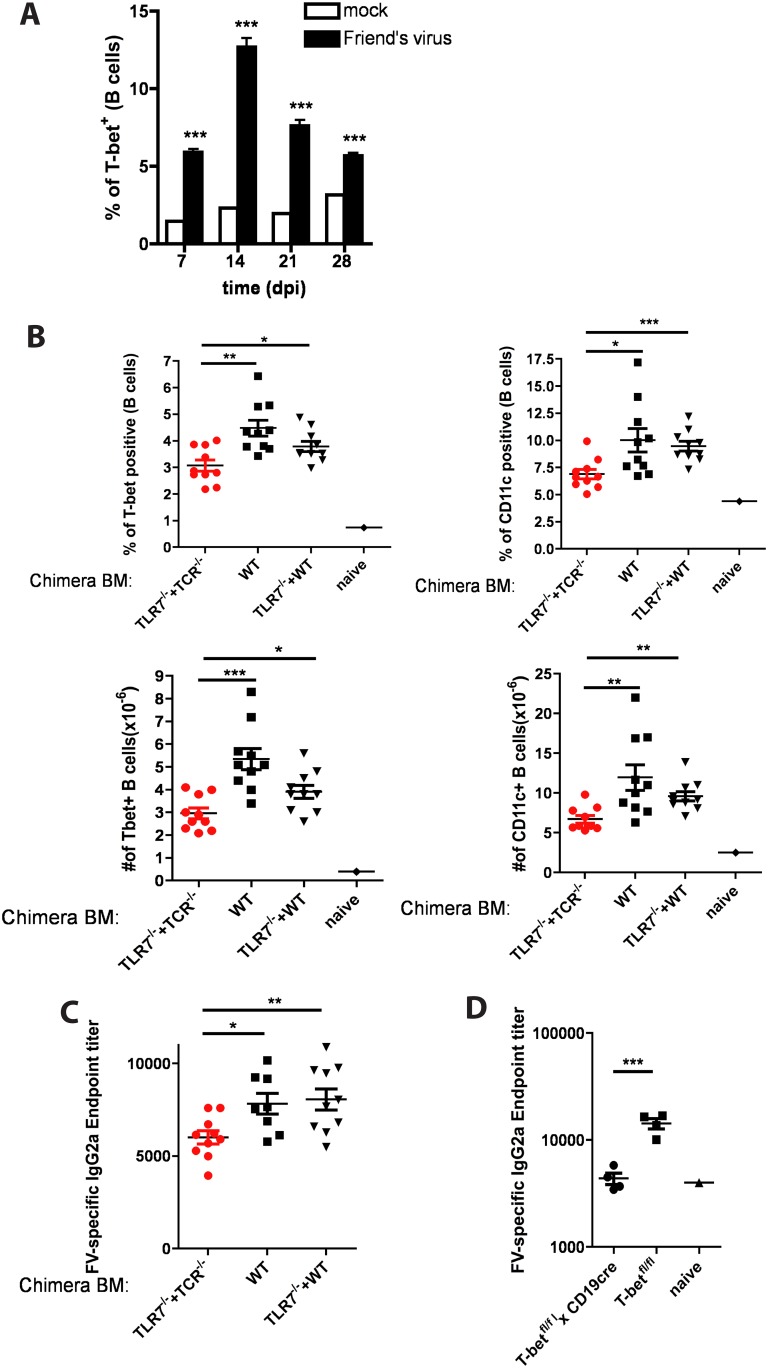
TLR7 expression in T cells is required for the accumulation of T-bet+ B cells and effective production of anti-viral IgG2a during Friend virus infection. **(A)** C57BL/6 mice (n = 5 per time point) were infected with 10^4^ SFFU of Friend virus (FV). Spleens were harvested on day 7, 14, 21 or 28 post infection. The presence of T-bet+ cells among B cells was assessed by FACS. Bar graphs represent percentage of T-bet+ cells among B cells. **(B, C)** Bone marrow chimera were constructed as described in Methods, such as in (TLR7^-/-^ + TCR^-/-^) mice only T cells completely lack TLR7 expression and the rest of the hematopoetic cells were 80% WT and 20% TLR7-/-. Bone marrow chimeras were infected with FV (as in **(A)**) and spleens and serum were harvested on 14 dpi percentage and numbers of T-bet+ or CD11c+ B cells is shown **(B).** Serum collected at 14dpi was assessed for the presence of anti-FV IgG2a by ELSIA **(C)**. **(D)** T-bet^fl/fl^, and T-bet^fl/fl^xCD19^CRE^ mice (n = 4 mice per group) were infected with FV as in (A). Serum was collected at 15 dpi and the presence of anti-FV IgG2a was assessed by ELISA. Bars represent the means +/- SEM. Data are representative of three or more independent experiments.

IgG2a is the predominant IgG isotype produced during Friend virus (FV) infection [29]. To check that this is accompanied, in FV infection, by the appearance of T-bet+ B cells, we infected C57BL/6 mice with the virus. As shown in in Fig 4A, we indeed detected T-bet+ B cells at 7–21 dpi with a peak in their numbers at day 14 in C57Bl/6 mice. The T-bet+ B cells co-expressed CD11c as has been previously reported for T-bet+ B cells in γHV68 infection [13] (data not shown).

To find out whether this phenomenon depended on TLR7 signaling in T cells, we generated (TLR7^-/-^+ TCRβ^-/-^) mixed bone marrow chimeras, in which all T cells were TLR7-/- and the rest of the hematopoietic cells were 80% WT and 20% TLR7-/-.

(TLR7^-/-^ + TCRβ^-/-^) and control chimeras were infected with FV. Their spleen cells were harvested and analysed at 14 dpi. The data indicate that mice with TLR7-deficient T cells had reduced percentages and numbers of T-bet+/CD11c+ B cells (Fig 4B) when compared with control mice, suggesting that TLR7 expression in T cells is required for efficient T-bet upregulation in B cells during FV infection. Serum levels of anti-viral IgG were measured to find out if the reduction in T-bet+ B cell numbers affected humoral immunity. As shown in Fig 4C, (TLR7^-/-^ + TCRβ^-/-^) mice had significantly reduced levels of anti-FV IgG2a. Anti-FV IgG2a was still apparent to some extent in the (TLR7^-/-^ + TCRβ^-/-^) mice, probably because TCR engagement of the TLR7^-/-^ FV specific T cells could still induce some IFNγ.

We confirmed that the reduction in T-bet+ B cells was the reason for the reduced anti-viral IgG2a production in these experiments by infecting T-bet^fl/fl^ x CD19^cre/wt^ mice with FV. As shown in Fig 4D, the results in these animals were similar: mice with a B-cell specific T-bet deletion produced significantly lower levels of anti-FV IgG2a than control animals did.

The mice in these experiments were all on the C57BL/6 background. Such mice are profoundly resistant to FV infection and, in all animals splenic proviral DNA levels were extremely low by day 14 pi. Thus we could not determine whether the absence of TLR7 signaling in T cells or T-bet expression in B cells affected FV titers at this time. However, the data shown here indicate that, during FV infection, T cells respond to TLR7 triggering and this response is necessary for efficient T-bet upregulation in B cells and their isotype switching to IgG2a. In the absence of TLR7 in T cells, the appearance of T-bet+/CD11c+ B cell and IgG2a isotype switching is significantly reduced.

## Discussion

In this report we show that subsets of memory T cells (CD4 effector and CD8 central memory) respond to TLR7/IL-12 stimulation by producing IFNγ. The idea that T cells could respond to TLR ligands used to be controversial. However, many recent studies have shown that some T cells express TLRs and respond accordingly to their ligands [3, 30–32]. Most of these studies were performed under conditions in which the T cells were also activated by ligation of their TCRs. However, one study showed that, in contrast to naïve T cells, human memory T cells could make small amounts of IFNγ in response to TLR ligation, although, even in this case, the response was very much improved by co-ligation of TCR [7]. Our demonstration here that TLR7 ligand in combination with IL-12 can induce enough IFNγ production by a subset of memory T cells to have consequences for, at least, B cell responses is therefore the first to show that this phenomenon may be more than an intellectual curiosity. Indeed, it suggests that this type of T cell activation could occur in the absence of antigen challenge and might occur chronically in animals and humans that do not efficiently clear TLR7 ligands, such as single stranded RNA released from dying cells, with consequences for the animal involved.

It was surprising that, as we have previously demonstrated, TLR7 was the only TLR ligand that generated IFNγ production by memory T cells in particular and by whole splenic cells in general [13]. This result raised the question how TLR7 differs from other TLRs in this particular process? So far we do not have an explanation for this phenomenon. None of the existing studies indicate elevated levels of *tlr7* or even *MyD88* gene expression in memory T cells (http://www.immgen.org) leaving us with suggestion that, perhaps, TLR7 signaling is differently regulated in memory T cells. It is also possible that different TLRs induce different amounts of IL-12 from splenic cells, which in turn is required for IFNγ production by memory T cells. All of these very important questions will be explored in the future.

It has been previously demonstrated by several groups that memory T cells can produce IFNγ in antigen-independent manner in response to IL-12/IL-18 [22, 23, 33]. The mechanism of IL-12/IL-18 synergy has recently been described indicating that IL-12 signaling leads to the generation of *infg* mRNA and IL-18-induced signaling is needed for the stabilization of this massage. Since both IL-18 and TLR7 signal via MyD88, it is possible that TLR7 plays similar role stabilizing *ifng* mRNA, leaving us with the same question: why TLR7 is the only TLR capable of IFNγ induction from memory T cells.

Others and we have shown that IFNγ induces T-bet expression and switching in B cells to production of immunoglobulin of the IgG2a isotypes [13, 24, 26]. It is fairly well known that, in mice, this antibody isotype effects the most efficient clearance of viruses, perhaps because it binds activating Fc receptors with the highest affinity [34]. Therefore our finding that, TLR7 ligands, via T cell production of IFNγ, induce switching to IgG2a most efficiently is not surprising. However, it is surprising that this phenomenon is manifested even in virus infected mice. In these animals virus specific CD4 and CD8 T cells are activated and might be expected to produce copious amounts of IFNγ, even in the absence of engagement of their endogenous TLR7. Such was not observed, however. In the absence of TLR7 in T cells, infection with FV induced significantly less IgG2a production. Thus our experiments underline the importance of the pathway we have uncovered, and explain more fully how the most effective antibody responses to virus are induced.

The findings reported here may be relevant to the design of vaccines in humans. At the moment it is not clear exactly which IgG isotypes clear virus most effectively in humans, nor how switch to such isotypes can be induced. If the results reported in this manuscript apply also to humans, they suggest that vaccines that contain a TLR7 agonist as an adjuvant might create the most appropriate types of immune response for virus clearance.

## Supporting Information

S1 Fig T cell specific deletion of MyD88 in MyD88flox/flox x LCK-cre mice. Splenocytes from MyD88flox/flox x LCK-cre (red line), MyD88flox/flox x LCK-wt (blue line) or MyD88KO (gray solid histogram) were stained for surface markers and intracellularly stained for MyD88. MyD88 expression on T cells or B cells is shown.
(PDF)

## References

[1] Iwasaki A, Medzhitov R. Toll-like receptor control of the adaptive immune responses. Nature immunology. 2004;5(10):987–95. Epub 2004/09/30. 10.1038/ni1112 . 15454922

[2] Reynolds JM, Dong C. Toll-like receptor regulation of effector T lymphocyte function. Trends in immunology. 2013;34(10):511–9. Epub 2013/07/28. 10.1016/j.it.2013.06.003 . 23886621

[3] Caramalho I, Lopes-Carvalho T, Ostler D, Zelenay S, Haury M, Demengeot J. Regulatory T cells selectively express toll-like receptors and are activated by lipopolysaccharide. The Journal of experimental medicine. 2003;197(4):403–11. Epub 2003/02/20. 10.1084/jem.20021633 12591899 PMC2193858

[4] Kabelitz D. Expression and function of Toll-like receptors in T lymphocytes. Current opinion in immunology. 2007;19(1):39–45. Epub 2006/11/30. 10.1016/j.coi.2006.11.007 . 17129718

[5] Ehlers M, Fukuyama H, McGaha TL, Aderem A, Ravetch JV. TLR9/MyD88 signaling is required for class switching to pathogenic IgG2a and 2b autoantibodies in SLE. The Journal of experimental medicine. 2006;203(3):553–61. Epub 2006/02/24. 10.1084/jem.20052438 16492804 PMC2118244

[6] Pasare C, Medzhitov R. Control of B-cell responses by Toll-like receptors. Nature. 2005;438(7066):364–8. Epub 2005/11/18. 10.1038/nature04267 . 16292312

[7] Gelman AE, Zhang J, Choi Y, Turka LA. Toll-like receptor ligands directly promote activated CD4+ T cell survival. Journal of immunology. 2004;172(10):6065–73. Epub 2004/05/07. 15128790 10.4049/jimmunol.172.10.6065 PMC2833313

[8] Bartholdy C, Christensen JE, Grujic M, Christensen JP, Thomsen AR. T-cell intrinsic expression of MyD88 is required for sustained expansion of the virus-specific CD8+ T-cell population in LCMV-infected mice. The Journal of general virology. 2009;90(Pt 2):423–31. Epub 2009/01/15. 10.1099/vir.0.004960-0 . 19141452

[9] Rahman AH, Cui W, Larosa DF, Taylor DK, Zhang J, Goldstein DR, et al MyD88 plays a critical T cell-intrinsic role in supporting CD8 T cell expansion during acute lymphocytic choriomeningitis virus infection. Journal of immunology. 2008;181(6):3804–10. Epub 2008/09/05. 18768833 10.4049/jimmunol.181.6.3804 PMC2835404

[10] LaRosa DF, Stumhofer JS, Gelman AE, Rahman AH, Taylor DK, Hunter CA, et al T cell expression of MyD88 is required for resistance to Toxoplasma gondii. Proceedings of the National Academy of Sciences of the United States of America. 2008;105(10):3855–60. Epub 2008/03/01. 10.1073/pnas.0706663105 18308927 PMC2268781

[11] Zhang Y, Jones M, McCabe A, Winslow GM, Avram D, MacNamara KC. MyD88 signaling in CD4 T cells promotes IFN-gamma production and hematopoietic progenitor cell expansion in response to intracellular bacterial infection. Journal of immunology. 2013;190(9):4725–35. Epub 2013/03/26. 10.4049/jimmunol.1203024 23526822 PMC3633622

[12] Schenten D, Nish SA, Yu S, Yan X, Lee HK, Brodsky I, et al Signaling through the adaptor molecule MyD88 in CD4+ T cells is required to overcome suppression by regulatory T cells. Immunity. 2014;40(1):78–90. Epub 2014/01/21. 10.1016/j.immuni.2013.10.023 . 24439266 PMC4445716

[13] Rubtsova K, Rubtsov AV, van Dyk LF, Kappler JW, Marrack P. T-box transcription factor T-bet, a key player in a unique type of B-cell activation essential for effective viral clearance. Proceedings of the National Academy of Sciences of the United States of America. 2013;110(34):E3216–24. Epub 2013/08/08. 10.1073/pnas.1312348110 23922396 PMC3752276

[14] Rubtsova K, Marrack P, Rubtsov AV. TLR7, IFNgamma, and T-bet: Their roles in the development of ABCs in female-biased autoimmunity. Cellular immunology. 2014 Epub 2014/12/30. 10.1016/j.cellimm.2014.12.002 . 25541140 PMC4380581

[15] Rubtsov AV, Rubtsova K, Fischer A, Meehan RT, Gillis JZ, Kappler JW, et al Toll-like receptor 7 (TLR7)-driven accumulation of a novel CD11c B-cell population is important for the development of autoimmunity. Blood. 2011;118(5):1305–15. Epub 2011/05/06. 10.1182/blood-2011-01-331462 21543762 PMC3152497

[16] Rubtsova K, Rubtsov AV, Cancro MP, Marrack P. Age-Associated B Cells: A T-bet-Dependent Effector with Roles in Protective and Pathogenic Immunity. Journal of immunology. 2015;195(5):1933–7. 10.4049/jimmunol.1501209 26297793 PMC4548292

[17] Smith DS, Guo K, Barrett BS, Heilman KJ, Evans LH, Hasenkrug KJ, et al Noninfectious retrovirus particles drive the APOBEC3/Rfv3 dependent neutralizing antibody response. PLoS pathogens. 2011;7(10):e1002284 Epub 2011/10/15. 10.1371/journal.ppat.1002284 21998583 PMC3188525

[18] Robertson SJ, Ammann CG, Messer RJ, Carmody AB, Myers L, Dittmer U, et al Suppression of acute anti-friend virus CD8+ T-cell responses by coinfection with lactate dehydrogenase-elevating virus. Journal of virology. 2008;82(1):408–18. Epub 2007/10/26. 10.1128/JVI.01413-07 17959678 PMC2224392

[19] Santiago ML, Montano M, Benitez R, Messer RJ, Yonemoto W, Chesebro B, et al Apobec3 encodes Rfv3, a gene influencing neutralizing antibody control of retrovirus infection. Science. 2008;321(5894):1343–6. Epub 2008/09/06. 10.1126/science.1161121 18772436 PMC2701658

[20] Maizel AL, Mehta SR, Ford RJ, Lachman LB. Effect of interleukin 1 on human thymocytes and purified human T cells. The Journal of experimental medicine. 1981;153(2):470–5. Epub 1981/02/01. 6787168 10.1084/jem.153.2.470 PMC2186072

[21] Rosenwasser LJ, Dinarello CA, Rosenthal AS. Adherent cell function in murine T-lymphocyte antigen recognition. IV. Enhancement of murine T-cell antigen recognition by human leukocytic pyrogen. The Journal of experimental medicine. 1979;150(3):709–14. Epub 1979/09/19. 314491 10.1084/jem.150.3.709 PMC2185642

[22] Nakahira M, Ahn HJ, Park WR, Gao P, Tomura M, Park CS, et al Synergy of IL-12 and IL-18 for IFN-gamma gene expression: IL-12-induced STAT4 contributes to IFN-gamma promoter activation by up-regulating the binding activity of IL-18-induced activator protein 1. Journal of immunology. 2002;168(3):1146–53. . 11801649 10.4049/jimmunol.168.3.1146

[23] Smeltz RB. Profound enhancement of the IL-12/IL-18 pathway of IFN-gamma secretion in human CD8+ memory T cell subsets via IL-15. Journal of immunology. 2007;178(8):4786–92. . 17404259 10.4049/jimmunol.178.8.4786

[24] Gerth AJ, Lin L, Peng SL. T-bet regulates T-independent IgG2a class switching. International immunology. 2003;15(8):937–44. Epub 2003/07/29. . 12882831 10.1093/intimm/dxg093

[25] Peng SL, Li J, Lin L, Gerth A. The role of T-bet in B cells. Nature immunology. 2003;4(11):1041; author reply Epub 2003/10/31. 10.1038/ni1103-1041a . 14586416

[26] Peng SL, Szabo SJ, Glimcher LH. T-bet regulates IgG class switching and pathogenic autoantibody production. Proceedings of the National Academy of Sciences of the United States of America. 2002;99(8):5545–50. Epub 2002/04/18. 10.1073/pnas.082114899 11960012 PMC122806

[27] Markine-Goriaynoff D, Coutelier JP. Increased efficacy of the immunoglobulin G2a subclass in antibody-mediated protection against lactate dehydrogenase-elevating virus-induced polioencephalomyelitis revealed with switch mutants. Journal of virology. 2002;76(1):432–5. Epub 2001/12/12. 10.1128/JVI.76.1.432-435.2002 11739710 PMC135718

[28] Coutelier JP, van der Logt JT, Heessen FW, Vink A, van Snick J. Virally induced modulation of murine IgG antibody subclasses. The Journal of experimental medicine. 1988;168(6):2373–8. Epub 1988/12/01. 3199074 10.1084/jem.168.6.2373 PMC2189165

[29] Halemano K, Guo K, Heilman KJ, Barrett BS, Smith DS, Hasenkrug KJ, et al Immunoglobulin somatic hypermutation by APOBEC3/Rfv3 during retroviral infection. Proceedings of the National Academy of Sciences of the United States of America. 2014;111(21):7759–64. Epub 2014/05/14. 10.1073/pnas.1403361111 24821801 PMC4040588

[30] Caron G, Duluc D, Fremaux I, Jeannin P, David C, Gascan H, et al Direct stimulation of human T cells via TLR5 and TLR7/8: flagellin and R-848 up-regulate proliferation and IFN-gamma production by memory CD4+ T cells. Journal of immunology. 2005;175(3):1551–7. Epub 2005/07/22. . 16034093 10.4049/jimmunol.175.3.1551

[31] Bendigs S, Salzer U, Lipford GB, Wagner H, Heeg K. CpG-oligodeoxynucleotides co-stimulate primary T cells in the absence of antigen-presenting cells. European journal of immunology. 1999;29(4):1209–18. Epub 1999/05/06. 10.1002/(SICI)1521-4141(199904)29:04<1209::AID-IMMU1209>3.0.CO;2-J . 10229088

[32] Gelman AE, LaRosa DF, Zhang J, Walsh PT, Choi Y, Sunyer JO, et al The adaptor molecule MyD88 activates PI-3 kinase signaling in CD4+ T cells and enables CpG oligodeoxynucleotide-mediated costimulation. Immunity. 2006;25(5):783–93. Epub 2006/10/24. 10.1016/j.immuni.2006.08.023 17055754 PMC2840381

[33] Yoshimoto T, Takeda K, Tanaka T, Ohkusu K, Kashiwamura S, Okamura H, et al IL-12 up-regulates IL-18 receptor expression on T cells, Th1 cells, and B cells: synergism with IL-18 for IFN-gamma production. Journal of immunology. 1998;161(7):3400–7. . 9759857

[34] Nimmerjahn F, Bruhns P, Horiuchi K, Ravetch JV. FcgammaRIV: a novel FcR with distinct IgG subclass specificity. Immunity. 2005;23(1):41–51. Epub 2005/07/26. 10.1016/j.immuni.2005.05.010 . 16039578

