# Supplementary material for: T Cell Production of IFNγ in Response to TLR7/IL-12 Stimulates Optimal B Cell Responses to Viruses
Source: PLoS One. 2016 Nov 23;11(11):e0166322. doi: 10.1371/journal.pone.0166322 (PMC5120817; doi:10.1371/journal.pone.0166322)
Supplement: S1 Fig — Splenocytes from MyD88flox/flox x LCK-cre (red line), MyD88flox/flox x LCK-wt (blue line) or MyD88KO (gray solid histogram) were stained for surface markers and intracellularly stained for MyD88. MyD88 expression on T cells or B cells is shown. (PDF) [file pone.0166322.s001.pdf]

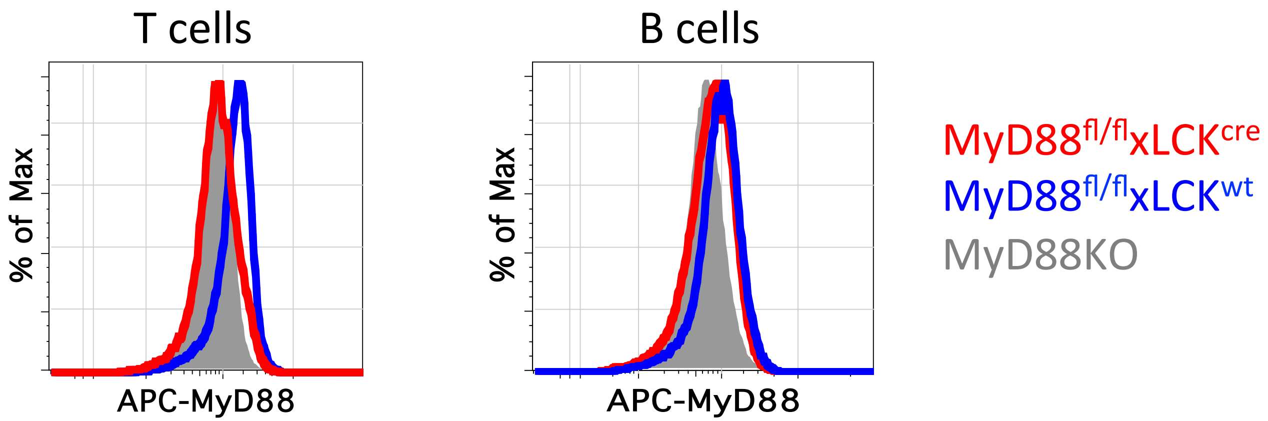

S1 Fig. T cell specific deletion of MyD88 in MyD88flox/flox x LCK-cre mice. Splenocytes from MyD88flox/flox x LCK-cre (red line), MyD88flox/flox x LCK-wt (blue line) pr MyD88KO (gray solid histogram) were stained for surface markers and intracellularly stained for MyD88. MyD88 expression on T cells or B cells is shown.

Rubtsova S1 Fig
